# Screening of Gestational Diabetes and Hypertension Among Antenatal Women in Rural West India

**DOI:** 10.5195/cajgh.2014.140

**Published:** 2014-12-08

**Authors:** Viral R. Dave, Bhavik M. Rana, Kantibhai N. Sonaliya, Suraj J. Chandwani, Samkit V. Sharma, Swati O. Khatri, Khalid M. Shaikh, Farida M. Hathiari

**Affiliations:** 1 Community Medicine Department, Gujarat Cancer Society (GCS) Medical College, Ahmedabad, Gujarat, India; 2 Gujarat Cancer Society (GCS) Medical College Hospital & Research Centre, Ahmedabad, Gujarat, India

**Keywords:** gestational diabetes, hypertension, screening, maternal mortality, perinatal mortality

## Abstract

**Background:**

Hypertension and gestational diabetes are among the leading causes of maternal and perinatal mortality, especially in rural areas of developing countries with meager health facilities. With early diagnosis and timely treatment, these adverse events can be decreased. The primary aim of this study was to implement a screening program for gestational diabetes and hypertension, and to assess risk factors associated with these conditions among antenatal women in the rural area of the Gujarat province in India.

**Methods:**

A cross–sectional study was conducted at one of the rural areas of Gujarat province in India. Following a random cluster sampling procedure, the village of Davas was selected. A multistage random sampling method was utilized, resulting in a sample of 346 antenatal women. Screening guidelines from the American Diabetes Association were followed for gestational diabetes screening.

**Results:**

The majority of antenatal mothers (55.50%) were between 21–25 years of age. 242 antenatal women were multigravida, and among them, 85.96% had institutional delivery at their last pregnancy. Of the total 346 women, 17.60% were prehypertensive. The prevalence of systolic hypertension was 1.40%, diastolic hypertension was 0.90%, and gestational diabetes was 1.73%.

**Conclusion:**

Socioeconomically upper class, a family history of hypertension, and BMI ≥ 25 were strong risk factors for hypertension during pregnancy and gestational diabetes. Health education should be made readily available to antenatal mothers by paramedical workers regarding symptoms of hypertension and gestational diabetes mellitus for early self identification.

Maternal mortality and perinatal mortality are two very important indicators of the developmental index of a country and have a significant impact on the population’s life expectancy. Hypertension (HTN) during pregnancy/pre-eclampsia and gestational diabetes are among the leading causes of maternal and perinatal morbidity and mortality, especially in rural areas of developing countries.^1^,^2^

The literature suggests that 10–15% of maternal mortality in developing countries is due to hypertensive disorders of pregnancy. 3,4 Various adverse effects of HTN in pregnancy include preterm delivery, intrauterine growth retardation, reduced birth weight, still birth, and perinatal mortality. 5,6 Early detection and prompt care are required to prevent adverse outcomes of pregnancy with HTN. For this reason, it is recommended to evaluate a woman’s risk for HTN at their first prenatal visit. In India, the National Health Programme of Reproductive and Child Health has stipulated routine screening of blood pressure (BP) for antenatal mothers at each visit, which should be implemented in conjunction with the assessment of maternal history of HTN symptoms.

Prevalence of gestational diabetes mellitus (GDM) in some ethnic groups ranges from 1 to 14% depending on screening method selection, diagnostic criteria, and population screened. GDM can negatively affect pregnancy and result in adverse perinatal outcomes such as macrosomia, birth trauma, shoulder dystocia, and higher rates of Cesarean section. 7 In India, screening is essential in all pregnant women, as Indian women have an eleven-fold increased risk 8 of developing glucose intolerance during pregnancy compared to Caucasian women, 9 which can be decreased with early screening, diagnosis, and treatment. It is generally accepted that women of Asian origin, especially ethnic Indians, are at a higher risk of developing GDM and subsequently type 2 diabetes. 10,11 The so-called Asian-Indian phenotype refers to certain unique clinical and biochemical abnormalities in Indians, which includes, but is not limited to, increased insulin resistance and greater abdominal adiposity. This phenotype makes Indians more prone to diabetes. 12

The objective of this study was to implement screening for gestational diabetes and HTN and to investigate risk factors associated with the development of these conditions among women in Gujarat province in India. The ultimate goal of this work was to minimize complications and reduce morbidity and mortality associated with gestational diabetes and HTN in high-risk groups.

## Methodology

After obtaining permission from the Institutional Ethical Committee at Gujarat Cancer Society (GCS) Medical College, Hospital & Research Centre, Ahmedabad, India, the present study was conducted at one of the rural areas of the Gujarat province in India. The study was conducted between March 2013 and June 2013 and utilized cross-sectional assessments. A multistage random cluster sampling method was utilized to obtain a sample of 346 antenatal women. After preparing a list of districts of Gujarat state in their ascending order as per their population, the Banaskantha district was randomly selected. Following random selection procedure, the taluka “Deesa” (administrative subunit of district) and the village of “Davas” were selected.

The Primary Health Centre (PHC) is a very basic medical setting, which provides health care services to rural populations. The PHC of Davas was contacted to ascertain a list of “anganwadi” discovered in the field practice area of PHC. Anganwadi is a part of the Indian public health-care system where basic health-care activities are carried out for antenatal and postnatal women, adolescent girls, and children less than 6 years of age. PHC in Davas covers a total population of 39,001 people and 15 other villages around Davas, as per the 2011 census. Anganwadi centers are a basic unit of the Integrated Child Development Scheme run by the government of India and provide health care services to antenatal and postnatal women, adolescent girls, and children less than 6 years of age. Monthly prenatal check ups is one of the key health services provided by these centers. A total of 346 antenatal women were registered with all anganwadis covered by Davas PHC. All were interviewed and after informed consent was taken from each participant, they were included in the study. A pretested and structured questionnaire on socio-demographic information, obstetric details, and outcome of screening tests was used for data collection. Various risk factors generally associated with gestational diabetes and HTN such as age, BMI (body mass index), education, occupation, socioeconomic class, and family history were also assessed using questionnaires.

The modified Prasad classification 13 was used for socioeconomic stratification, which is commonly used on the Indian subcontinent. This classification system divides the community into five different classes based on per capita income of family and taking into consideration All India Consumer Price Index declared by the Labour Bureau, Government of India at the time of study. 14

For screening of gestational diabetes, guidelines of American Diabetes Association 15 were followed, which state that: a fasting plasma glucose level > 126 mg/dl (7.0 mmol/l) or casual plasma glucose > 200 mg/dl (11.1 mmol/l) meets the threshold for diagnosis of diabetes, if confirmed on a subsequent day and precludes need for any glucose challenge. Following the above-mentioned guidelines, random blood sugar levels (RBS) were measured using a standard glucometer (Glucosign @ Accubiotech). Participants with RBS > 200 mg/day were re-screened on the following day with a fasting blood sugar.

Blood pressure was classified per the following criteria: 16 normal if < 120 systolic BP and < 80 diastolic BP, prehypertension if 120–139 systolic BP or 80–89 diastolic BP, Stage I HTN if 140–159 systolic BP or 90–99 diastolic BP, and Stage II HTN if systolic BP ≥ 160 or diastolic BP ≥ 100. A standard sphygmomanometer was used to measure BP. All BP measurements were taken in sitting position, and on finding BP higher than normal, a recording was repeated immediately and also on the next day to ensure that measurements were accurate. Conservative recording of the lowest reading was evaluated in the data analysis. Data entry and data analysis were performed using SPSS software. Risk factors for HTN and diabetes were analysed by bi-variate analysis along with the use of odds ratios.

## Results

Table 1 presents sociodemographic data of study participants at baseline. The majority of antenatal mothers (55.50%) were 21–25 years of age. It is noteworthy that 21.10% of pregnant women were ≤ 20 years of age while 2.89% of participants were > 30 years aged. 60.98% of expectant mothers were illiterate, and only 0.87% had education at secondary school level, while none of them had completed any secondary education. Most of participant females were housewives (63.58%), while 34.97% of mothers were engaged in strenuous labor activities such as farming, construction, etc. Based on modified Prasad classification for socioeconomic class, the majority of women belonged to lower socioeconomic classes. 39.60% of women were from class IV and 42.77% belonged to socioeconomic class V. 7.23% of antenatal women had a BMI ≥ 25.

Table 2 describes obstetric profiles of study participants. 69.94% of antenatal women were multigravida, while 30.06% were primigravida. In multigravida participants, most deliveries were institutional (85.96%) while 14.04% of mothers had their last delivery without medical supervision. Of this 14.04% of mothers, the majority had home delivery; however, some delivered at their work place, including farms. 11.57% had their deliveries conducted by traditional birth attendants (TBA) known as “dai” in India, while 2.47% of deliveries were conducted by other attendants (including relatives, neighbors, and quacks).

**Figure 1:**
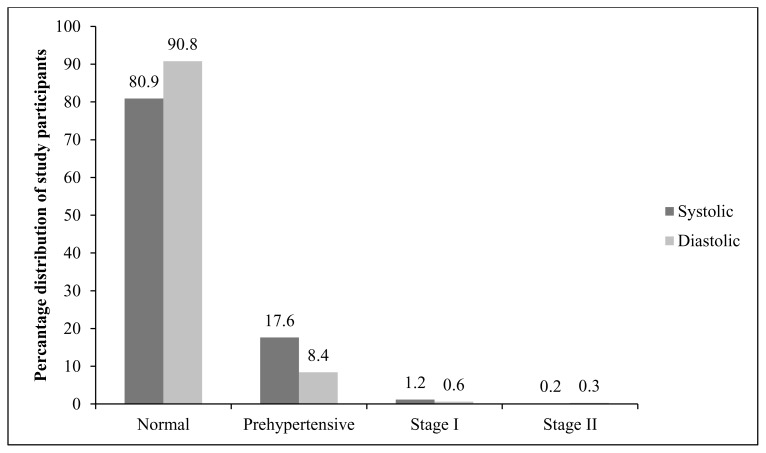
Distribution of stages of hypertension among antenatal women at screening

Figure 1 shows the distribution of BP among antenatal women in the study at screening. It suggests that 80.90% of study participants had systolic BP within normal range, while 17.60% were prehypertensive. 1.20% and 0.20% had stage I and stage II systolic BP HTN, respectively. Diastolic BP distribution was somewhat different. 90.80% had normal diastolic BP. 8.40% were prehypertensive, while 0.60% and 0.30% had stage I and II diastolic BP HTN, respectively.

Table 3 suggests the role of various risk factors associated with HTN, using odds ratio based on bi-variate analysis. The risk factors evaluated were age, BMI ≥ 25, lower socioeconomic class, occupation, education and family history positive for HTN. Upper socioeconomic class and positive family history of HTN were significantly (*p* < 0.05) associated with HTN among study participants.

Random blood sugar levels were checked for screening of GDM as per guidelines mentioned in methodology. The screening yielded 1.73% antenatal mothers had their RBS levels more than 200 mg/dl on two occasions. None of them were known cases of diabetes, while 98.27% had their RBS within normal range.

The role of various risk factors associated with gestational diabetes, using odds ratio based on bi-variate analysis is described in Table 4. It shows that among various risk factors such as age, BMI ≥ 25, upper socioeconomic class, occupation, education, and family history of HTN, only a BMI of ≥ 25 was significantly (*p* < 0.05) associated with GDM among study participants.

## Discussion

The present study found the prevalence of systolic HTN was 1.40%, diastolic HTN was 0.90%, and GDM was 1.73%. Upper socioeconomic class and positive family history of HTN were significantly associated with HTN. While analyzing other risk factors, only BMI ≥ 25 was significantly associated with GDM among study participants.

A study conducted by Yadav et al. 17 found a prevalence of HTN as high as 8.70% among females of less than 40 years of age. However, the sample included females from an urban colony of high-income residents among the general population, which could be responsible for the high prevalence. While the government is expending a considerable amount of funds as well as manpower to decrease maternal and perinatal mortality, it appears that residents of remote rural and tribal areas may not be taking advantage of some of the screening and early diagnosis practices.

The overall prevalence of diabetes in the western region of India was found to be 3.70%; 18 however, in this study, all age groups as well as all genders were included in this study. A previous study 19 conducted in Punjab region of India showed a prevalence of systolic HTN at 4.45% and diastolic HTN at 4.20% in a sample of 1,000 pregnant females, while the present study found a prevalence of systolic HTN was only 1.40% and diastolic HTN was 0.90%. The differences between this study and the above mentioned study could be attributed to a larger sample size from both rural and urban areas of the Punjab state used in the previous study.

Sayeed et al. 20 found in their study at Bangladesh a crude prevalence of systolic and diastolic HTN was 6.80% and 5.40%, respectively, possibly due to the inclusion of a higher aged sample. Bener and Saleh 21 in their study at Qatar found maternal age > 30, increased BMI, previous abortion, lack of antenatal care, and physical activity were significantly associated with an increased risk of Pregnacy Induced Hypertension (PIH). In the current study, upper socioeconomic class and positive family history of HTN were found to be associated with HTN, which is comparable to other similar studies. 20,21

In the present study, the prevalence of GDM was found to be 1.73%. Kalra et al. 22 found a prevalence of GDM among the study population was 6.60%, while Gupta et.al. 23 in the Jammu region of India found a prevalence of GDM was 3.05%. These data reveal a wide variation in the prevalence of gestational diabetes in India. A previous study 24 conducted in Haryana, North India, showed 7.10% prevalence, while another study 25 conducted in South India revealed prevalence of 17.80% women in urban, 13.80% in semi urban, and 9.90% in rural population of gestational diabetes. Sayeed et al. 20 in Bangladesh found the prevalence of diabetes was 6.80% according to fasting blood glucose (FBG) guideline values. In the present study, we found that only BMI ≥ 25 was significantly associated with GDM, while Sheshiah et al. 25 found that age ≥ 25 years, BMI ≥ 25 kg/m^2^, and positive family history of diabetes were significantly associated with GDM. Rajput et al. 24 found that socioeconomic status above upper middle class and Kalra et al. 22 found that family history of diabetes mellitus, age ≥ 25 years, past history of GDM, and BMI ≥ 25 kg/m^2^ were significantly associated with GDM group. Rajput et al. 24 found that socioeconomic status above upper middle class was associated significantly with GDM in their findings. Kalra et al. 22 found that a family history of diabetes mellitus, age ≥ 25 years, past history of GDM, and BMI ≥ 25 kg/m^2^ were significantly associated with GDM group in their study.

55.49% of participants in the present study were 21–25 years of age, and 60.98% were illiterate. In a similar study by Rajput et al. 24 58.20% were 21–25 years of age, while only 4.90% were illiterate. Literacy rate in the present study was low as compared to national average (65.46%) and Gujarat state average (70.73%) for females. 26 The reason may be due to the fact that the selected district was in a remote area with tribal vicinity. In the same study, Rajput et al. 24 found that 8.20% of participants had a BMI ≥ 25 while in current study it was 7.20%.

## Limitations of the Study

This investigation had one main limitation. Though most research recommends Oral Glucose Tolerance Test (OGTT) as a method of choice for screening of diabetes, this method was not possible to implement in the context in this study. OGTT is supposed to be performed under clinical observation with laboratory set up. It also requires multiple blood draws for which the subject needs to be contacted more than 2 times and requires longer time duration (3–4 hours). As the current study is a cross sectional study with limited resources at remote rural area, the necessary time, human resource and financial resources were limiting factors for conducting GTT.

## Strengths of the Study

As there are very limited number of studies conducted in the past involving pregnant women of rural India, this study can contribute significantly and provide a glimpse about health conditions of antenatal women in rural population of India. The current study also provided an opportunity to contact study subjects and identify some previously undetected at risk women who were referred for further evaluation and management.

## Conclusion & Recommendations

The present study found prevalence of systolic HTN was 1.40%, diastolic HTN was 0.90%, and GDM was 1.73% among study population in the rural area of Gujarat province, India. Overall, the prevalence of gestational diabetes and HTN in the Gujarat state should be evaluated in the future with a multicenter study and larger sample size. Incidence and prevalence rate of any specific disease is the basis for making prevention strategies at national level of health planning. A proper disease registry should be created for monitoring gestational diabetes and pregnancy induced HTN. Public health education should be made readily available to antenatal mothers by paramedical workers regarding alarming symptoms of HTN and GDM for early self identification. Adopting a healthy life style and monitoring blood pessure and sugar levels are keys for prevention of HTN and diabetes in India and globally.

## Figures and Tables

**Table 1:** Baseline characteristics of study participants from the Davas village

Characteristics	Number of Participants (%)
Age (years)	
≤ 20	73 (21.10)
21 – 25	192 (55.49)
26 – 30	71 (20.52)
31 – 35	9 (2.60)
≥ 36	1 (0.29)
Education	
Illiterate	211 (60.98)
Primary	132 (38.15)
Secondary and above	3 (0.87)
Occupation	
Housewife	220 (63.58)
Laborer	121 (34.97)
Other	5 (1.45)
Socioeconomic class	
I	3 (0.87)
II	18 (5.20)
III	40 (11.56)
IV	137 (39.60)
V	148 (42.77)
BMI (Kg/M2)	
< 18.5	68 (19.65)
18.5 – 25	253 (73.12)
≥ 25	25 (7.23)

**Table 2:** Obstetric profile of study participants

Characteristics	Number of Participants (%)
Gravida	
Primigravida	104 (30.06)
Multigravida	242 (69.94)
Last delivery conducted (n = 242)	
Institutional deliveries	208 (85.96)
Traditional birth attendants	28 (11.57)
Other	6 (2.47)
Mode of last delivery (n = 242)	
Full term normal delivery	202 (83.47)
Premature delivery	7 (2.89)
Miscarriage	2 (0.83)
Abortion	27 (11.16)
Cesarian section	4 (1.65)

**Table 3:** Odds ratio for risk factors found to be associated with systolic hypertension

Risk factors	Odds ratio	95% Confidence interval		*p-value*

		Lower	Upper	
Age > 25 years	0.81	0.08	7.37	0.60
BMI ≥ 25	3.29	0.35	30.62	1.25
SE Upper Class (I + II)*	7.77	1.27	47.60	0.03
Housewife	0.14	0.01	1.26	0.13
Education (Illiterate)	0.15	0.01	1.41	0.08
Family history*	2.89	0.32	25.83	0.02

*Note*.

* denotes significance with a *p* < 0.05.

**Table 4:** Odds ratio for risk factors found to be associated with gestational diabetes

Risk factors	Odds ratio	95% Confidence interval		*p-value*

		Lower	Upper	
Age > 25 years	1.66	0.30	9.26	0.38
BMI ≥ 25*	5.36	0.53	53.94	0.01
SE Upper Class (I + II)	1.53	0.73	3.21	0.46
Housewife	1.14	0.20	6.35	0.28
Education (Illiterate)	1.29	0.23	7.14	0.81
Family history	1.78	0.27	8.13	0.09

*Note*.

* denotes significance with a *p* < 0.05.
